# Variation of salinity and nitrogen concentration affects the pentacyclic triterpenoid inventory of the haloalkaliphilic aerobic methanotrophic bacterium *Methylotuvimicrobium alcaliphilum*

**DOI:** 10.1007/s00792-021-01228-x

**Published:** 2021-04-18

**Authors:** Alexmar Cordova-Gonzalez, Daniel Birgel, Andreas Kappler, Jörn Peckmann

**Affiliations:** 1 Institut für Geologie, Centrum für Erdsystemforschung und Nachhaltigkeit, Universität Hamburg, Hamburg, Germany; 2 Geomikrobiologie, Zentrum für Angewandte Geowissenschaften, Universität Tübingen, Tübingen, Germany

**Keywords:** Aerobic methanotrophs, Nitrate, Salinity, Pentacyclic triterpenoids, Bacteriohopanepolyols, Tetrahymanol

## Abstract

The occurrence and activity of aerobic methanotrophs are influenced by environmental conditions, including pH, temperature, salinity, methane and oxygen concentrations, and nutrient availability. Aerobic methanotrophs synthesize a variety of lipids important for cell functions. However, culture-based experiments studying the influence of environmental parameters on lipid production by aerobic methanotrophs are scarce. Such information is crucial to interpret lipid patterns of methanotrophic bacteria in the environment. In this study, the alkaliphilic strain *Methylotuvimicrobium alcaliphilum* was cultivated under different salinities and different nitrate concentrations to assess the effect of changing conditions on the inventory of pentacyclic triterpenoids. The results indicate that hopanoid abundance is enhanced at lower salinity and higher nitrate concentration. The production of most pentacyclic triterpenoids was favored at low salinity, especially for aminotriol. Interestingly, 3-methyl-aminotetrol and tetrahymanol were favored at higher salinity. Bacteriohopanepolyols (BHPs), particularly aminotriol and 3-methyl-aminotriol, increased considerably at higher nitrate concentrations. Four novel *N*-containing BHPs—aminodiol, 3-methyl-aminodiol, and isomers of aminotriol and 3-methyl-aminotriol—were identified. This study highlights the significance of environmental factors for bacterial lipid production and documents the need for cultivation studies under variable conditions to utilize the full potential of the biomarker concept.

**Supplementary Information:**

The online version contains supplementary material available at 10.1007/s00792-021-01228-x.

## Introduction

Aerobic methanotrophic bacteria are an important methane sink due to their ability to utilize methane as the sole carbon and energy source, using the key enzyme monooxygenase (Hanson and Hanson 1996; Semrau et al. 2010). Bacterial aerobic methane oxidation (MOx) contributes significantly to the withdrawal of methane released from anoxic sediments and soils, which otherwise could accumulate in the atmosphere as a severe greenhouse gas. Therefore, MOx is essential for methane consumption (Hanson and Hanson 1996; Sherry et al. 2016). Aerobic methanotrophs occur in terrestrial, freshwater, and marine ecosystems, preferably at oxic-anoxic interfaces. Aerobic methanotrophic bacteria are microaerophilic, using oxygen as electron acceptor and methane as carbon and energy source (Boetius and Wenzhöfer 2013; Bessette et al. 2017). Generally, aerobic methanotrophs are divided into two major groups, belonging to the Gammaproteobacteria (Type I and Type X methanotrophs) and Alphaproteobacteria (Type II methanotrophs), which differ in physiology, chemotaxonomy, internal ultrastructure, carbon assimilation pathways, and other biochemical aspects (Bowman 2006).

Type II methanotrophic bacteria are restricted to terrestrial environments such as peats, soils, and lakes. Type I methanotrophic bacteria are more versatile, also dwelling in the marine realm (e.g., Knief 2015). Generally, the occurrence and activity of aerobic methanotrophic bacteria are influenced by environmental conditions, including pH, temperature, salinity, methane and oxygen concentrations, and nutrient availability (Semrau et al. 2010; Knief 2015; Sherry et al. 2016). Among these factors, the effect of inorganic nitrogen on aerobic methanotrophs has been widely studied, particularly for agricultural soils, where ammonium- or nitrate-based fertilizers may influence methane oxidation and affect the global methane budget (Noll et al. 2008). Unfortunately, results of culture studies on the effect of inorganic nitrogen addition on methanotrophic activity are not in agreement with reports demonstrating either inhibition (Dunfield and Knowles 1995) or stimulation (Bodelier et al. 2000; Bodelier and Laanbroek 2004) of methane oxidation. Although the link between nitrogen availability and methane consumption is still unclear, it is assumed that the effect of nitrogen is dependent on the structure of the in situ methanotrophic community (Hoefman et al. 2014), since aerobic methanotrophs have shown metabolic variability regarding nitrogen assimilation on both genus and species level. The nitrogen metabolism of various aerobic methanotrophs strains is diverse, including cometabolic oxidation of ammonia (Nyerges and Stein 2009), nitrate reduction to nitrite (Bowman et al. 1993), detoxification of hydroxylamine and nitrite (Nyerges and Stein 2009; Nyerges et al. 2010), and fixation of atmospheric nitrogen, the latter known from few strains only (Hoefman et al. 2014; Tays et al. 2018). Among the various genera of aerobic methanotrophs, the nitrogen metabolism seems to be strain-specific (Hoefman et al. 2014). Nonetheless, gammaproteobacterial methanotrophs are preferentially stimulated by nitrogen amendments (Bodelier et al. 2000) and alphaproteobacterial methanotrophs cope better with nitrogen limitation than Gammaproteobacteria, perhaps due to their ability to fix dinitrogen (Nyerges and Stein 2009).

Another important factor influencing the activity of aerobic methanotrophs is salinity. Sherry et al. (2016) analyzed the response of a methanotroph community in estuarine sediments to salinity change and documented an inverse correlation of decreasing methane oxidation rates with increasing salinity. MOx communities at salinities higher than 35 g/l were dominated by Type I *Methylomicrobium* species, closely related to the halo-/alkalitolerant methanotroph *Methylotuvimicrobium alcaliphilum*, which lives at normal marine and higher salinities (Orata et al. 2018). Intriguingly, none of the sequences identified were affiliated with known marine methanotrophs (Sherry et al. 2016).

Among the most specific capabilities of aerobic methanotrophs is their ability to synthesize a unique lipid biomarker inventory. Apart from fatty acids with characteristic double bond positions (e.g., Hanson and Hanson 1996), this group of bacteria is known as a producer of a variety of source-specific pentacyclic (hopanoids) and tetracyclic triterpenoids (steroids) (e.g., Bouvier et al. 1976; Rohmer et al. 1984; Talbot and Farrimond 2007; Banta et al. 2015; Rush et al. 2016; Wei et al. 2016; Cordova-Gonzalez et al. 2020). The cyclic triterpenoids are known to be crucial for cell functions such as cell growth and survival (Kannenberg and Poralla 1999; Welander et al. 2009; Welander and Summons 2012). Aerobic methanotrophs are among the few bacteria capable to express their ability to produce significant amounts of 3-methyl hopanoids (Welander and Summons 2012) and bacteriohopanepolyols (BHPs) with terminal amino groups and three to five hydroxyl groups (amino BHPs), or a combination of both (Zundel and Rohmer 1985; Talbot et al. 2003). Amino BHPs are used by aerobic methanotrophs to cope with environmental stress (Welander and Summons 2012; Kulkarni et al. 2013). Previous studies have investigated the influence of temperature, pH, and nitrogen metabolism on bacterial hopanoid production. For example, contents of BHPs in *Bacillus acidocaldarius*, a thermoacidophilic bacterium, increased with increasing temperature, but only moderately with decreasing pH (Poralla et al. 1984), while a psychrotolerant strain of the aerobic methanotroph *Methylomonas methanica* revealed an opposite trend with respect to temperature, showing a decrease of hopanoid contents with increasing temperature (Jahnke et al. 1999). Strains of *Methylovulum psychrotolerans*, an aerobic psychrotolerant methanotroph, also showed a decrease in hopanol contents, specifically tetrafunctionalized BHPs and diplopterol, with increasing temperature, along with an increase in penta- and hexafunctionalized BHPs (Bale et al. 2019). Osborne et al. (2017) have studied the effect of varying temperatures on BHP production in a sedimentary methanotrophic community from the environment. These authors demonstrated a temperature dependence of aminopentol production, but not for aminotetrol or aminotriol. Nalin et al. (2000) have reported slightly increased hopanoid contents under nitrogen-replete (NH_4_^+^) conditions for several nitrogen-fixing actinomycete *Frankia* strains. Even though some studies exist, our knowledge on the influence of environmental parameters on lipid production by methanotrophic bacteria is still limited, being based on only a few culture experiments (Jahnke et al. 1999; Talbot et al. 2001; Cordova-Gonzalez et al. 2020). Often the interpretation of environmental samples is still challenging due to the lack of culture experiments under conditions close to those of the respective environments. More cultivation experiments are needed to evaluate possible unknown adaptations of aerobic methanotrophs strains in response to changing environmental conditions. Complementarily, experiments using uncultured strains in micro- and mesocosms (Sherry et al. 2016; Osborne et al. 2017) need to be performed to close the gap between the biomarker patterns of cultures and environmental samples.

Here, we use cultivation experiments to assess whether changes in nitrate availability and salinity affect the composition and abundance of pentacyclic triterpenoids produced by the Type I methanotroph *Methylotuvimicrobium alcaliphilum*, a haloalkaliphilic strain, which has been studied previously for its triterpenoid inventory (Banta et al. 2015; Rush et al. 2016; Cordova-Gonzalez et al. 2020). We report the occurrence of novel BHPs in *M. alcaliphilum* and modifications of its lipid inventory at varying salinities and nitrate concentrations. This approach will help to understand the limitations of aerobic methanotrophy in haloalkaline environments and will foster interpretation of biomarker patterns observed in the environment and the rock record.

## Materials and methods

*Methylomicrobium alcaliphilum* 20Z (now *Methylotuvimicrobium alcaliphilum*; Orata et al. 2018) strain was obtained from the Leibniz Institute DSMZ–German Collection of Microorganisms and Cell Cultures. The cultivation experiments were performed in the Geomicrobiology group at the University of Tübingen, Germany. *M. alcaliphilum* was first isolated from surface sediments of highly alkaline soda lakes in Russia (Kalyuzhnaya et al. 2008).

### Culture conditions

*M. alcaliphilum* strains were grown in 100 ml serum bottles filled with 10 ml of the DSMZ recommended *Methylomicrobium* medium (DSMZ 1180) containing 30 g NaCl, 0.20 g MgSO_4_ × 7 H_2_O, 0.02 g CaCl_2_ × 2 H_2_O and 1 g KNO_3_, in 1000 ml distilled water. The pH was adjusted to 8.5 and cultures were incubated at 28 °C and shaken at 200 rpm. The initial gas-mixing ratio was adjusted at methane:air 1:1 (*v*/*v*). Four different culture conditions were applied: standard growth conditions (3% NaCl, 0.1% or 10 mM KNO_3_), low salinity (1% NaCl), high salinity (8.7% NaCl), and high nitrate concentration (1% or 100 mM KNO_3_). Cells were harvested by centrifugation at 4000 rpm for 10 min when entering the stationary phase and freeze-dried for further analyses. Cell growth was monitored by measuring the optical density at 600 nm on a Thermo Scientific Multiskan Go spectrophotometer.

### Extraction and derivatization

Harvested cells were freeze-dried and gently ground, then extracted with dichloromethane (DCM)/methanol (MeOH) (3:1, *v*/*v*) by successive ultrasonication to produce the total lipid extract (TLE) according to Cordova-Gonzalez et al. (2020). An aliquot of the TLE was acetylated by adding acetic acid anhydride/pyridine (1:1 *v*/*v*) to the dried TLE at 60 °C for one hour and left at room temperature overnight. The solvent mixture was carefully dried with a gentle stream of N_2_. Hopanoids with ≤ 30 carbons and tetrahymanol were measured by gas chromatography–flame ionization detector (GC–FID) and coupled gas chromatography–mass spectrometry (GC–MS). For analysis of BHPs, the acetylated aliquots of TLE were measured by means of high-performance liquid chromatography–mass spectrometry (HPLC–MS). Tetrafunctionalized BHPs (aminotriols) were additionally measured on GC–MS with a high temperature column (see section “Gas chromatography and high-performance liquid chromatography”). A second aliquot of the underivatized TLE was treated with periodic acid and sodium boron hydride to transform BHPs in GC-amenable hopanols (Rohmer et al. 1984). Briefly, after periodic acid cleavage, tetrafunctionalized BHPs (e.g., aminotriol and bacteriohopanetetrol) were converted to C_32_ 17β(H),21β(H)-hopanol (bishomohopanol), pentafunctionalized BHPs (e.g., aminotetrol and bacteriohopanepentol) produce C_31_ 17β(H),21β(H)-hopanol (homohopanol), and hexafunctionalized BHPs (e.g., aminopentol) yield C_30_ 17β(H),21β(H)-hopanol (hopanol). The resulting hopanols were acetylated as described above.

### Gas chromatography and high-performance liquid chromatography

The acetylated TLEs were analyzed by GC–FID for quantification, using a Thermo Scientific Trace 1300 Series, and by GC–MS for identification, using a Thermo Scientific Trace GC Ultra coupled to a Thermo Scientific DSQ II mass spectrometer at the University of Hamburg, Germany. A programmed temperature vaporizer (PTV) was used for injection in both devices, the carrier gases were hydrogen for GC–FID and helium for GC–MS. Compounds were separated using an Agilent HP-5 MS UI fused silica capillary column (30 m length, 0.25 mm i.d., 0.25 μm film thickness). The GC temperature program was 50 °C (3 min) to 230 °C at 15 °C/min (2 min), 230–325 °C at 6 °C/min, 25 min isothermal. The identification by GC–MS was based on GC retention times and comparison of mass spectra with published data. Internal standards (1-nonadecanol and 5α-cholestane) with known concentrations were added prior to extraction for quantification. BHPs were analyzed as their BHP-cleaved hopanols under the same conditions. High temperature gas chromatography coupled with mass spectrometry (HTGC–MS) was performed on the same machines using a Zebron ZB-5HT capillary column (15 m length, 0.32 mm i.d., 0.10 µm film thickness). The GC temperature program was 50 °C (3 min) to 260 °C at 15 °C/min (0 min), 260–350 °C at 10 °C/min, 30 min isothermal.

High-performance liquid chromatography–mass spectrometry (HPLC–MS) analyses were performed using a Varian MS Workstation 6.91 HPLC system coupled to a Varian 1200L triple quadrupole mass spectrometer, equipped with a reversed-phase Phenomenex Kinetex® EVO C_18_ 100 Å column (150 mm length, 2.1 mm i.d., 2.6 μm particle size) and a security guard column cartridge of the same material. The program used was 0.14 ml/min at 35 °C with 100% *A* (0–3 min) to 100% *B* (at 30 min); isocratic (to 40 min), returning to starting conditions over 5 min and stabilizing for 5 min (where *A* = 90% MeOH/10% water and *B* = 59% MeOH/40% propan-2-ol/1% water). The MS instrument was equipped with an atmospheric pressure chemical ionization (APCI) source operated in positive ion mode. The detection was achieved at a peak width of 1.0 amu (scan time 2.5 s) and the mass scan range was set to *m/z* 200–1200. The acetylated extracts were injected in MeOH/propan-2-ol (3:2 *v*/*v*) and a known amount of internal standard (5α-pregnane-3β,17β,20β-triol) was added prior to analysis for a semi-quantitative estimate of the concentration of BHPs. Compounds were identified by comparison with published data and relative retention time. To verify the elution time of the regular aminotriol, peaks in the samples were compared with those in samples of *Desulfovibrio* strains (Blumenberg et al. 2006), run at the same conditions.

**Fig. 1:**
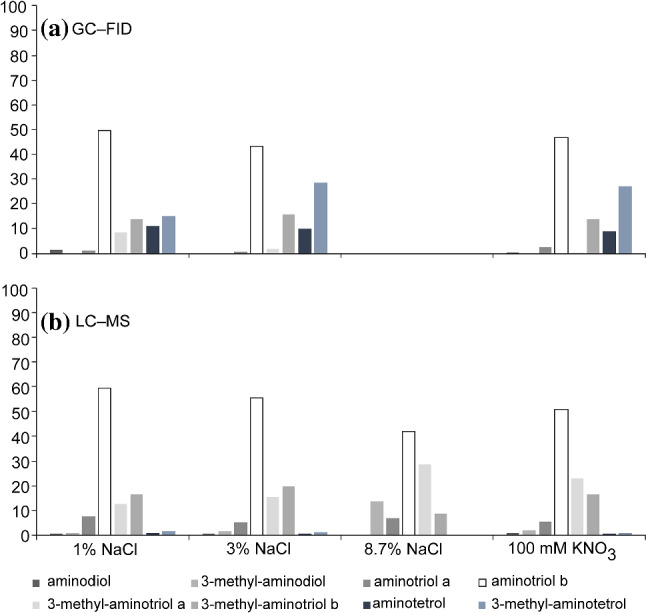
Comparison of quantification of BHPs of *M. alcaliphilum* as **a** hopanol products after periodic acid cleavage using GC–FID and **b** intact hopanols using LC–MS reflecting different salinities and nitrate concentration. GC–FID data for experiments at 8.7% NaCl are not shown

BHPs were measured as intact BHPs by means of HPLC–MS and as periodic acid cleavage products (Fig. 1a, b). GC–MS analyses of periodic acid cleavage products (Fig. 1) showed a considerably higher contribution of aminotetrol (**V**; see Appendix for roman numbers) and 3-methyl-aminotetrol (**VI**) than in HPLC–MS, suggesting and underestimation in the latter technique. In general, tetrafunctionalized BHPs (aminotriols **III** and 3-methyl-aminotriols **IV**) were more abundant in LC–MS measurements than in GC–FID measurements of the respective hopanol products. Pentafunctionalized BHPs (aminotetrol **V** and 3-methyl-aminotetrol **VI**) were less abundant in LC–MS measurements than their corresponding hopanol products measured by GC–FID. Similar discrepancies have been reported for tetrafunctionalized BHPs by van Winden (2011) and were attributed either to underestimation of pentafunctionalized compounds due to LC–MS response factors or to oversight of unknown pentafunctionalized BHPs in the LC–MS analyses. Other studies have shown that BHP contents measured with HPLC–MS are 50–100% higher than those measured with GC–FID (Sessions et al. 2013), although the reasons remain unclear. In view of these discrepancies, the BHP contents are reported herein as their periodic acid cleavage products only. Moreover, since standards were not available for each functionalized hopanoid, quantification of BHPs with HPLC–MS could introduce a large error, while GC–FID provides the most accurate quantitation assuming a uniform response factor (Jorgensen et al. 1990), particularly for compounds within the same class.

## Results and discussion

### Novel N-containing BHPs

**Fig. 2:**
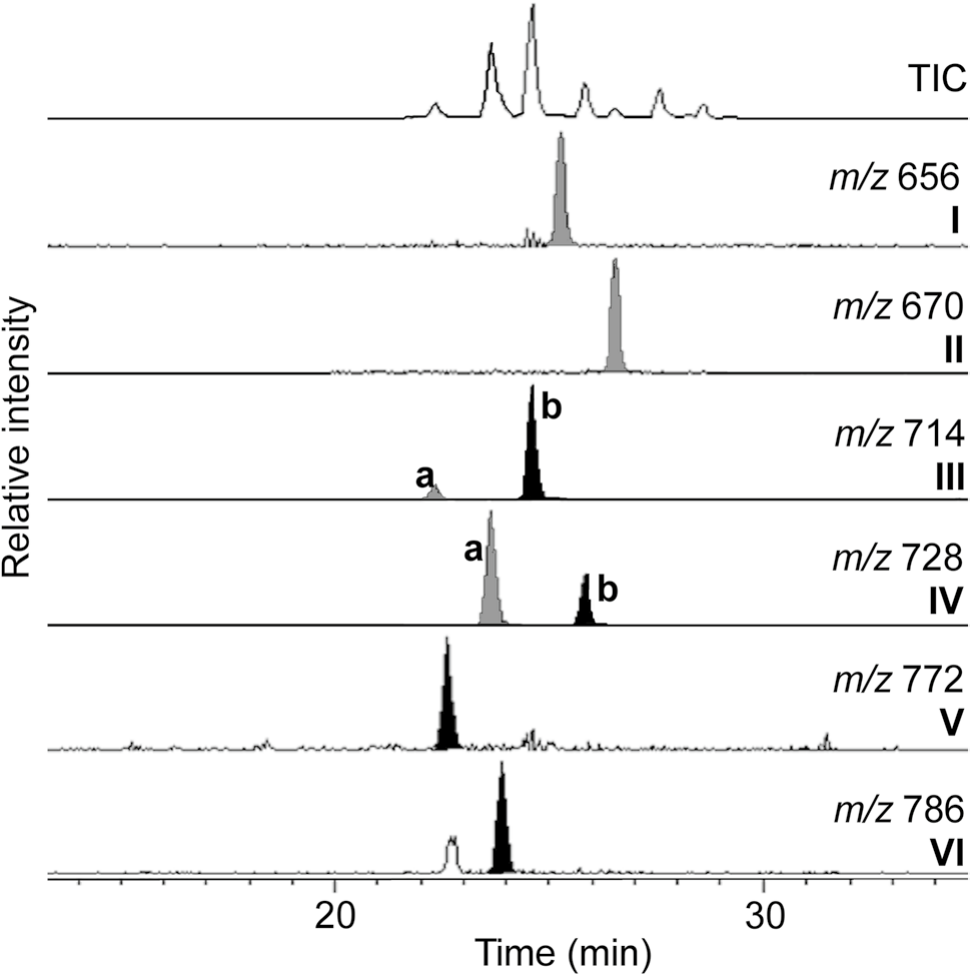
Partial HPLC–MS chromatograms (13–35 min) showing BHPs in the acetylated total lipid extract of *M. alcaliphilum* grown at 3% salinity and 100 mM KNO_3_. Novel BHPs are indicated in gray

**Fig. 3:**
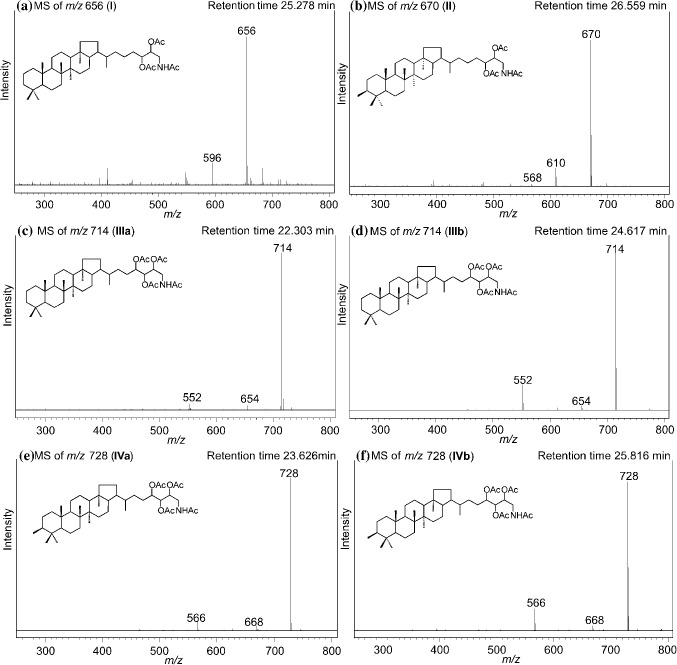
HPLC–MS spectra of acetylated **a** aminodiol **I**, **b** 3-methyl-aminodiol **II**, isomers of **c**, **d** aminotriol **IIIa,b** and **e**, **f** 3-methyl-aminotriol **IVa,b** of *M. alcaliphilum*

The inventory of lipid biomarkers of the Type I methanotroph *M. alcaliphilum* has been previously described by Banta et al. (2015), Rush et al. (2016), and Cordova-Gonzalez et al. (2020), although under different culturing conditions than those chosen in this study. Most abundant BHPs in *M. alcaliphilum* cultures are aminotriol (**IIIb**), 3-methyl-aminotriol (**IVb**), aminotetrol (**V**), and 3-methyl-aminotetrol (**VI**; Figs. 2, 3).

In addition to the amino-BHPs previously reported for *M. alcaliphilum* (**IIIb**, **IVb**, **V**, and **VI**; Figs. 2, 3), the HPLC–MS analysis revealed the presence of four novel components with base peaks at *m/z* 656 (acetylated; BHP-656 **I**), *m/z* 670 (acetylated; BHP-670 **II**), *m/z* 714 (**IIIa**) and *m/z* 728 (**IVa**), respectively ([M + H]^+^; Figs. 2, 3). These compounds are tentatively identified as novel *N*-containing BHPs on the basis of the even *m/z* value of the base peak ion (cf. Talbot et al. 2016).

The LC mass spectrum of BHP-656 (**I**; Fig. 3a) includes a minor peak at *m/z* 596, indicating a loss of 60 Da (i.e., acetylated OH group, CH_3_COOH). Compound BHP-656 **I** elutes later than aminotriol **IIIb**, suggesting a less polar compound on the reversed-phase HPLC column (see section “Gas chromatography and high-performance liquid chromatography”). The peak in question was tentatively identified as aminodiol. Directly after BHP-656 **I** elutes the compound BHP-670 **II**. The mass spectrum of BHP-670 **II** (Fig. 3b**)** includes other minor peaks at *m/z* 610 and *m/z* 568, indicating a neutral loss of 60 Da (i.e., acetylated OH group, CH_3_COOH) followed by a neutral loss of 42 Da (i.e., partial loss of acetylated OH group, COCH_2_), respectively. The 14 Da difference suggests non-methylated (**I**) and methylated (**II**) homologues, with the presence of a methyl group at C-3 in the latter, since methylation at this position is typical of Type I methanotrophs (Talbot et al. 2003).

As mentioned above, two new peaks with *m/z* 714 (**IIIa**) and *m/z* 728 (**IVa**) were identified, respectively. Both peaks **IIIa** and **IVa** were eluting earlier than the commonly found BHPs aminotriol **IIIb** and 3-methyl-aminotriol **IVb**, respectively (see Fig. 2). The mass spectrum of *m/z* 714 **IIIa** (Fig. 3c) is almost identical to the mass spectrum of regular aminotriol **IIIb** (Fig. 3d) with ([M + H]^+^  = *m/z* 714 and minor peaks at *m/z* 654, *m/z* 612 and *m/z* 552 representing neutral losses of 60 Da (i.e., acetylated OH group) followed by losses of 42 Da (i.e., loss of partial fragment of acetylated OH group) and 60 Da, respectively. However, the peak at *m/z* 552 ([M + H-2CH_3_COOH-COCH_2_]^+^) in the MS spectrum of compound **IIIa** is less prominent than in the MS spectrum of regular aminotriol **IIIb** and the association ion at *m/z* 774 ([M + H + 60]^+^) (sensu Talbot et al. 2001) is absent.

**Fig. 4:**
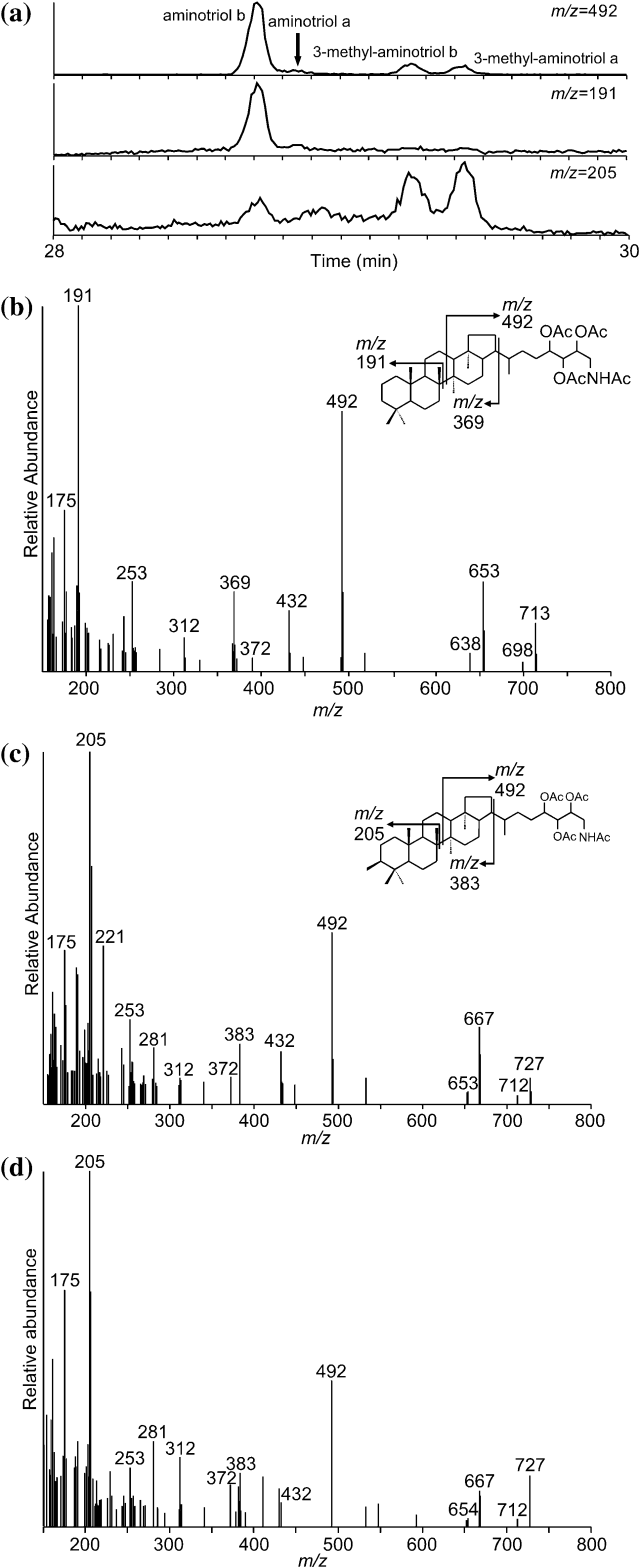
**a** HTGC–MS partial chromatograms (*m/z* 492; *m/z* 191; *m/z* 205) of acetylated total lipid extract of *M. alcaliphilum*. **b** EI mass spectrum of an acetylated isomer of aminotriol b **IIIb.** **c** EI mass spectra of acetylated 3-methyl-aminotriol b **IVb**. **d** EI mass spectra of acetylated 3-methyl-aminotriol a **IVa**

Analogous to *m/z* 714 peaks, the spectrum of *m/z* 728 **IVa** (Fig. 3e) is similar to the mass spectrum of regular 3-methyl-aminotriol **IVb** (Fig. 3f) with ([M + H]^+^  = *m/z* 728) and minor peaks at *m/z* 668 (loss of 60 Da; i.e., acetylated OH group), *m/*z 626 (loss of 42 Da; i.e., loss of a partial fragment of an acetylated OH group) and *m/z* 566 (neutral loss of 60 Da). In the MS spectrum of **IVa**, the peak at *m/z* 566 is less prominent than in the MS spectrum of regular 3-methyl-aminotriol **IVb** and the association ion at *m/z* 788 ([M + H + 60]^+^) is absent. Compounds **IIIa** and **IVa** have been tentatively assigned as stereoisomers of aminotriol **IIIb** and 3-methyl-aminotriol **IVb**, respectively. Unfortunately, the similarity between MS spectra of the respective isomers did not allow discrimination of the stereochemistry of the compounds. The relative abundances of compounds **IIIa** and **IVa** of all measured BHPs are 5% and 16%, respectively (relative abundance in the HPLC runs for experiments with *M. alcaliphilum* run at standard conditions). However, co-elution of compounds **IIIa** with aminotetrol **V** and compound **IVa** with 3-methyl-aminotetrol **VI** complicated the assessment of the peak areas. The production of compounds **IIIa** and **IVa** as a consequence of fragmentation in the source of the HPLC–MS from the known aminotriol **IIIb** and 3-methyl-aminotriol **IVb** was discarded as two isomers of aminotriol and 3-methyl-aminotriol were also found in HTGC–MS analyses (Fig. 4a). The order of elution on HTGC–MS is reversed compared to HPLC analyses due to measurement on a reversed-phase column on the HPLC–MS.

HTGC–MS analyses of intact BHPs were used to further confirm the presence and possible structure of compounds **IIIa** and **IVa**. The tentative identification of aminotriol stereoisomers using HTGC–MS was based on their molecular masses and expected fragmentation patterns from Sessions et al. (2013) and comparison with retention time and mass spectra of aminotriol **IIIb** from a co-injected extract of *Desulfovibrio* strain BSS (cf. Blumenberg et al. 2006). The extracted *m/z* 191 chromatogram showed two peaks, eluting at slightly different retention times (Fig. 4a), with M^+^ at *m/z* 713 (Fig. 4b; corresponding to [M + H]^+^ at *m/z* 714 in HPLC–MS). Diagnostic ions include *m/z* 713 (M^+^), *m/z* 492 (ring D + E + side chain), *m/z* 369 (ring A–E), *m/z* 191 (ring A + B), and other fragments representing losses of acetylated groups such as *m/z* 432 (492–60), *m/z* 372 (492–60 × 2), *m/z* 312 (492–60 × 3), and *m/z* 653 (713–60). The full GC–MS spectrum for the novel aminotriol **IIIa** is not displayed, due to the low concentration of the compound. BHP-656 **I** and BHP-670 **II** were not found in HTGC–MS analyses, most likely due to the even lower concentrations of these compounds, since the sensitivity of measurements with HPLC–MS are 50–100% higher than those measured with GC–MS. This phenomenon is possibly caused by more severe analyte loss during GC–MS analyses (cf. Sessions et al. 2013). Peaks at *m/z* 653 and *m/z* 668 were observed in HTGC–MS analyses and interpreted as degradation products of aminotriol **III** and 3-methyl-aminotriol **IV**, when injected on a PTV or split/splitless injector and run on the ZB-5HT column (see Sessions et al. 2013; Eickhoff et al. 2014).

The identification of 3-methylhomologs of aminotriol is based on molecular mass and changes in major MS fragmentation from *m/z* 191 to *m/z* 205. The *m/z* 205 chromatogram showed two peaks, similar as for desmethylated aminotriol (Fig. 4a). The GC–MS spectra of the two stereoisomers of 3-methyl-aminotriol (**IVa** and **IVb**) showed fragments characteristic of the hopanoid rings (*m/z* 205 and *m/z* 383; Fig. 4c, d), and a major peak at *m/z* 492 (ring D + E + side chain). Diagnostic fragments resulting from the loss of one or more acetic acid groups, as described above for aminotriol **IIIb**, were apparent in the mass spectrum of 3-methyl-aminotriols **IVa,b** (*m/z* 667, 432, 372, 312). The identification of the proposed stereoisomers of aminotriol and 3-methyl-aminotriol in both analyses, HTGC–MS and HPLC–MS, helps to exclude an artificial production of the novel described amino-BHPs. Moreover, HTGC–MS spectra confirmed the structure of aminotriol **III** and 3-methyl-aminotriol **IV** under EI conditions.

The stereochemistry of both identified isomers of aminotriol **IIIa,b** and 3-methyl-aminotriol **IVa,b** could not be clarified with HTGC–MS and HPLC–MS either, due to low contents. Typically, the spectra of the biological stereoisomers 17β(H),21β(H) of common hopanes exhibit a stronger response for the ring D + E + side chain fragment (*m/z* 492) than for the ring A + B fragment (*m/z* 191 for aminotriol or *m/z* 205 for 3-methyl-aminotriol), although this reasoning does not necessarily extend to all related hopanoids (Peters et al. 2004). In this study, all isomers identified in HTGC–MS showed a ring A + B fragment dominating over the D + E + side chain fragment, however, no evidence was found for the presence of isomers with another configuration than the biological 17β(H),21β(H) stereoisomer.

**Fig. 5:**
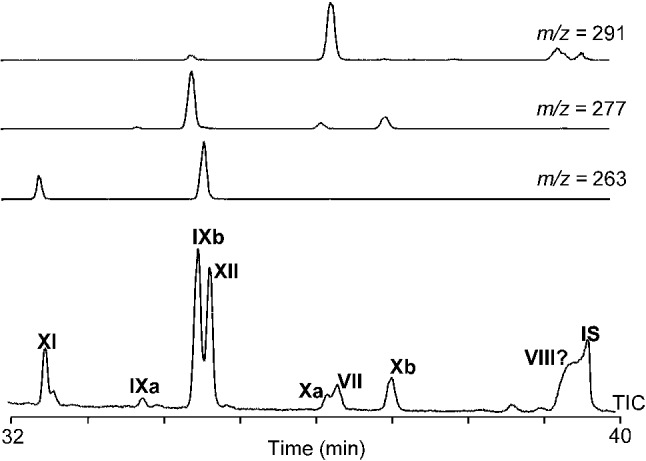
Partial GC–MS chromatograms (*m/z* 291; *m/z* 277; *m/z* 263) of hopanol products obtained after periodic acid cleavage. Aminotetrol yielded homohopanol **XI**, 3-methyl-aminotetrol yielded 3-methyl-homohopanol **XII**, aminotriols yielded bishomohopanols **IX**, 3-methyl-aminotriols yielded 3-methyl-bishomohopanols **X**, and aminodiol yielded tentatively identified trishomohopanol **VII**. **VIII = **tentatively identified 3-methyl-trishomohopanol (hopanol product of 3-methyl-aminodiol) coeluted with the internal standard (IS)

In the cultures of *M. alcaliphilum*, the two isomers of aminotriol **IIIa,b** and 3-methyl-aminotriol **IVa,b** are probably best explained by isomerization at the chiral centers of the side chain. The isomers are most likely C-32 epimers, since the two isomers of aminotriol and two isomers of 3-methyl-aminotriol were present in most samples after periodic acid cleavage (Fig. 5). Periodic acid cleavage of aminotriol produces C_32_ 17β(H),21β(H)-hopanol **IX** (bishomohopanol). Consequently, isomers at positions C-33, C-34, and C-35 would have been lost after cleavage, resulting in one single isomer of C_32_ 17β(H),21β(H)-hopanol. The same holds true for 3-methyl-aminotriol. The formation of ββ isomers of aminotriol and methylated homologues (at C-2 position) have been previously described in cultures of the purple non-sulfur bacterium *Rhodopseudomonas palustris* TIE-1, although only after artificial maturation (170 °C, 120 MPa; see Eickhoff et al. 2014). In great contrast to the artificial production of isomers in experiments with *R. palustris* TIE-1, the stereoisomers in cultures of *M. alcaliphilum* were produced at optimum temperature and pressure conditions and not as a product of degradation experiments.

The production of BHP isomers in bacteria is common. Isomers of bacteriohopanetetrol (aminotriol equivalent *N*-free BHP) have been previously reported in a variety of bacterial cultures (e.g., *Komagataeibacter xylinus*, *Frankia sp.*, *Methylocella palustris*, *‘Ca. Brocadia sp.’*, *‘Ca. Scalindua profunda’* and *‘Ca. Scalindua brodeae’*; Rush et al. 2014; Schwartz-Narbonne et al. 2020).

### Effect of salinity and nitrate concentration on growth rates

*M. alcaliphilum* is a haloalkaliphilic aerobic methanotrophic bacterium belonging to the Type I methanotrophs. *M. alcaliphilum* strains grow optimally at NaCl concentration of 3% (Kalyuzhnaya et al. 2019), and a nitrogen source (KNO_3_) concentration of 10 mM (0.1%) is recommended. Here, *M. alcaliphilum* was grown under four different conditions, standard conditions (3% salinity, 10 mM KNO_3_), high nitrate concentration (100 mM KNO_3_), low salinity (1%) and high salinity (8.7%). All conditions were chosen arbitrarily considering the range of conditions tolerated by the strain (Kalyuzhnaya et al. 2008). Growth rates (Table 1, Eq. 1) were highest for the experiments with low salinity and high nitrate concentration, while the experiment at high salinity yielded the slowest growth rate. Lag phases (Supplementary figure S1) were also shorter for experiments grown at low salinity, high nitrate concentrations, and standard conditions, while they were longer for the experiment with high salinity, suggesting poor growth or a long period of adaptation to the condition (Tays et al. 2018). Growth rates (*α*) were calculated from two data points on the growth curve (covering an interval of logarithmic growth) using the formula:

1 $$\alpha = \frac{\mathrm{ln}\left(\frac{{N}_{T}}{{N}_{0}}\right)}{\left({t}_{T- {t}_{0}}\right)},$$

where *t* is time, and *N* is the cell number (defined by OD_600nm_) at time *t* (Tays et al. 2018).

**Table 1:** Growth rates for each condition tested

Experiment	Growth rate (1/h)
3% NaCl, 100 mM KNO_3_	3.90*E*−02
1% NaCl, 10 mM KNO_3_	3.17*E*−02
8.7% NaCl, 10 mM KNO_3_	1.32*E*−02
3% NaCl, 10 mM KNO_3_	2.08*E*−02

### Effect of salinity and nitrate concentration on the pentacyclic triterpenoid inventory

Pentacyclic triterpenoids synthesized by *M. alcaliphilum* comprise (3-methyl **IV**) aminotriol **III**, (3-methyl **VI**) aminotetrol **V**, (3-methyl **XX**) tetrahymanol **XIX**, and minor amounts of (3-methyl **XVIII**) diplopterol **XVII**, (3-methyl **XV**) diploptene **XIII**, and (3-methyl **XVI**) hop-21-ene **XIV** (Tables 2 and 3; contents of pentacyclic triterpenoids of single experiments are provided in Supplementary File). However, hop-21-ene **XIV** (and its 3-methylhomologue **XVI**), as well as other hopenes such as, hop-17(21)-ene, and neohop-13(18)-ene (Eickhoff et al. 2013a), apparently formed by dehydration of diplopterol **XVII** during injection on the GC–MS via PTV or split/splitless injection and are not produced by the bacteria (Sessions et al. 2013). Given the natural variation typical for culture experiments and the fact that the experiments of this study were prepared from different batches of freshly prepared starter cultures, a certain variability in the data is to be expected. Still, the standard deviation for some of the compounds is too high, precluding a meaningful comparison. Furthermore, cell numbers (OD_600_) were determined for one replicate under each culture condition only; it is therefore possibly that samples were harvested during different growth phases.

**Table 2:** Contents of pentacyclic triterpenoids in cultures of *M. alcaliphilum* grown at different salinities

Compound	1% NaCl (*n* = 3)		3% NaCl (*n* = 3)	
	Content (µg/g dw)	SD	Content (µg/g dw)	SD
diploptene **XIII**	28	12	21	6
hop-21-ene **XIV**	16	6	10	3
3-me-diploptene **XV**	6	3	5	2
3-me-hop-21-ene **XVI**	2	0	3	1
diplopterol **XVII**	72	39	40	22
3-me-diplopterol **XVIII**	15	1	12	5
tetrahymanol **XIX**	55	2	50	14
3-me-tetrahymanol **XX**	2	1	4	2
aminodiol **I**^a^	12	4	0	0
aminotriol a **IIIa**^a^	9	1	3	5
aminotriol b **IIIb**^a^	420	243	172	34
3-me-aminotriol a **IVa**^a^	**71**	28	**8**	6
3-me-aminotriol b **IVb**^a^	117	70	63	20
aminotetrol **V**^a^	93	49	40	8
3-me-aminotetrol **VI**^a^	127	52	114	26
Sum pentacyclic triterpenoids	1045	413	544	88
3-me-diploptene/diploptene	0.20	0.03	0.23	0.04
3-me-diplopterol/diplopterol	0.18	0.04	0.29	0.04
3-me-tetrahymanol/tetrahymanol	0.03	0.01	0.09	0.01
3-me-aminotriol/aminotriol b	0.28	0.04	0.37	0.04
3-me-aminotetrol/aminotetrol	1.35	0.49	2.87	0.13
3-methylhopanoids/hopanoids	0.43	0.10	0.77	0.03

^a^BHPs were measured as products of periodic acid cleavage (aminotetrol yielded homohopanol, aminotriols yielded bishomohopanols, aminodiol yielded trishomohopanol). Bold values show statistically significant difference with respect to standard growing conditions (3% NaCl) using unpaired *t* test (*α* < 0.05). *SD* standard deviation, *dw* dry weight

**Table 3:** Contents of pentacyclic triterpenoids in cultures of *M. alcaliphilum* grown with varying amounts of nitrate

Compound	10 mM KNO_3_ (*n* = 3)		100 mM KNO_3_ (*n* = 3)	
	Content (µg/g dw)	SD	Content (µg/g dw)	SD
diploptene **XIII**	21	6	18	7
hop-21-ene **XIV**	10	3	9	3
3-me-diploptene **XV**	5	2	8	7
3-me-hop-21-ene **XVI**	3	1	2	0
diplopterol **XVII**	40	22	47	0
3-me-diplopterol **XVIII**	12	5	23	21
tetrahymanol **XIX**	50	14	136	92
3-me-tetrahymanol **XX**	4	2	19	19
aminodiol **I**^a^	0	0	2^b^	1
aminotriol a **IIIa**^a^	**3**	5	**27**	14
aminotriol b **IIIb**^a^	**172**	34	**467**	159
3-me-aminotriol a **IVa**^a^	8	6	4^b^	2
3-me-aminotriol b **IVb**^a^	**63**	20	**140**	30
aminotetrol **V**^a^	40	8	90	69
3-me-aminotetrol **VI**^a^	114	26	267	200
sum pentacyclic triterpenoids	544	88	1239	608
3-me-diploptene/diploptene	0.23	0.04	0.43	0.25
3-me-diplopterol/diplopterol	0.29	0.04	0.49	0.36
3-me-tetrahymanol/tetrahymanol	0.09	0.01	0.14	0.05
3-me-aminotriol/aminotriol b	0.37	0.04	0.30	0.05
3-me-aminotetrol/aminotetrol	2.87	0.13	2.97	0.14
3-methyhopanoids/hopanoids	0.77	0.03	0.87	0.11

^a^BHPs were measured as products of periodic acid cleavage (aminotetrol yielded homohopanol, aminotriols yielded bishomohopanols, aminodiol yielded trishomohopanol). Bold numbers indicate statistically significant differences to standard growth conditions (10 mM KNO_3_) using unpaired *t* test (*α* < 0.05). *SD* standard deviation, *dw* dry weight

^b^Average of 2 samples

For consistency with the section ‘Novel N-containing BHPs’, BHPs are referred to by the name of the intact compounds, even if the reported contents correspond to the measurement of hopanol cleavage products (see also “Materials and methods” section). The periodic acid cleavage procedure yielded C_32_ 17β(H),21β(H)-hopanol (bishomohopanol **IX**) from tetrafunctionalized BHPs and C_31_ 17β(H),21β(H)-hopanol (homohopanol **XI**) from pentafunctionalized BHPs. Since the only tetrafunctionalized and pentafunctionalized BHPs produced by *M. alcaliphilum* are aminotriol **III** and aminotetrol **V**, respectively, the bishomohopanol **IX** and homohopanol **XI** (and their respective methyl homologues) derived exclusively from these compounds (Fig. 5). Minor amounts of 17β(H),21β(H)-33-hopanol (trishomohopanol **VII**) in samples grown at high nitrate and low salinity are interpreted as cleavage product of aminodiol (BHP-560 **I**).

**Fig. 6:**
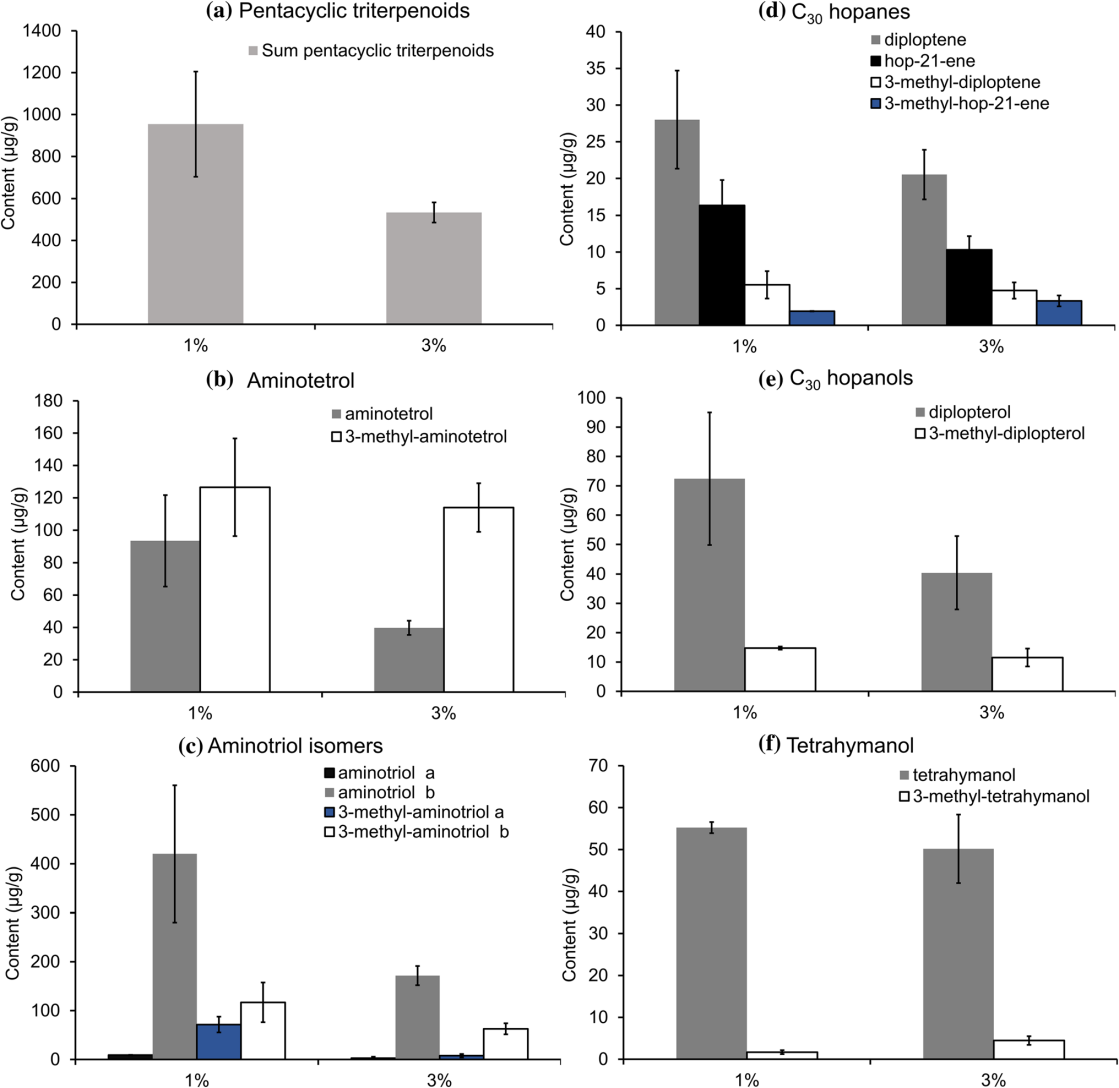
Distribution of pentacyclic triterpenoids in response to variations in salinity (1% and 3%): **a** total pentacyclic triterpenoids, **b** aminotetrol, **c** aminotriol isomers, **d** C_30_ hopanes, **e** C_30_ hopanols, **f** tetrahymanol. BHPs were measured as products of periodic acid cleavage (aminotetrol yielded homohopanol, aminotriols yielded bishomohopanols). Error bars represent standard errors for *n* = 3 replicates

In experiments at different salinities, the total amount of pentacyclic triterpenoids decreased with increasing salinity (Fig. 6a; Table 2), suggesting that hopanoid production in *M. alcaliphilum* is higher at lower salinities. The overall contents of pentacyclic triterpenoids produced at 8.7% NaCl (maximum salinity tolerance, Kalyuzhnaya et al. 2008) could not be determined since contents were too low (no measurements on the GC-FID were possible). However, measurement of BHPs on the HPLC–MS (Fig. 1) revealed that under high salinity conditions *M. alcaliphilum* produced less hopanoids than under standard (3%) and low salinity (1%) conditions. Experiments at 8.7% NaCl also gave the slowest growth rates (Table 1), indicating reduced activity of *M. alcaliphilum* under such conditions. Reduced activity of *M. alcaliphilum* at higher salinity is consistent with experiments performed by Sherry et al. (2016) on sediments from the River Tyne estuary, UK, hosting a diverse community of methanotrophic bacteria, where methanotrophic activity and methane oxidation rates decreased with increasing salinity.

**Fig. 7:**
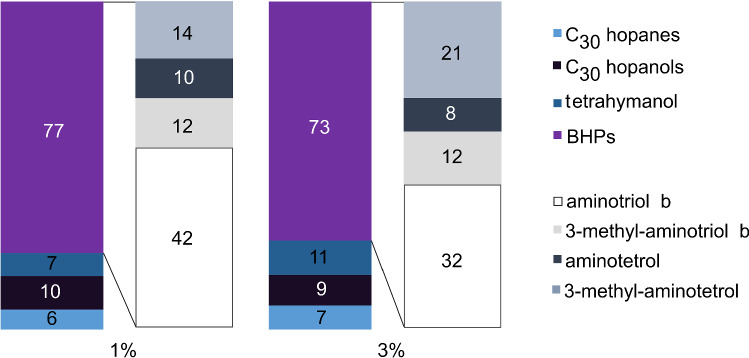
Relative abundance in percent of main pentacyclic triterpenoids of *M. alcaliphilum* in response to variations in salinity (1% and 3%). BHPs were measured as products of periodic acid cleavage (aminotetrol yielded homohopanol, aminotriol yielded bishomohopanol). Novel compounds (aminodiol, aminotriol a, and 3-methyl-aminotriol a) were excluded from calculations due to low contents

In our experiments, most hopanoids were produced at the lowest salinity (1% NaCl; Fig. 6), with the highest contents of aminotriols **IIIa,b** and the corresponding 3-methylhomologues **IVa,b** (Fig. 6c), and 3-methyl-aminotetrol **VI**, and tetrahymanol **XIX** with lowest contents (Fig. 6b, f). According to statistical analysis (unpaired *t* test; *p* values from *t* tests are provided in Supplementary File), only 3-methyl-aminotriol a **IIIa** varied significantly between different culture conditions. More relevant than changes of the absolute content of a specific hopanoid are changes of the proportion of this molecule to the considered class of compounds at varying salinities (Fig. 7); i.e., the relative abundance of a compound. The relative abundances of C_30_ hopanoids and C_30_ hopanols were similar under the two conditions tested. Tetrahymanol **XIX** showed an increase of 4% in experiments with 3% NaCl, while the proportion of BHPs decreased by 3%. Tetrahymanol—and its degradation product gammacerane—are commonly used as biomarkers for stratification of the water column; these compounds were also suggested to indicate hypersaline conditions in stratified water bodies (Sinninghe Damsté et al. 1995; Peters et al. 2004; Banta et al. 2015). Our results confirm that an increase of salinity indeed favors the synthesis of tetrahymanol **XIX** at the expense of hopanoids. Even though aerobic methanotrophic bacteria are not the only known producers of tetrahymanol (Rashby et al. 2007; Eickhoff et al. 2013b; Werne et al. 2002), all tetrahymanol producers seem to have in common that they live at oxic-anoxic interfaces or high salinities.

The results of this study indicate that low salinities favor the formation of BHPs (Fig. 7), especially the formation of the regular aminotriol b **IIIb**, which showed a relative increase of 10% at 1% NaCl. Interestingly, 3-methyl-aminotetrol **VI**, which is among the most specific biomarkers of aerobic methanotrophic bacteria (Talbot et al. 2014), showed a relative increase of 7% in the 3% NaCl experiments, consistent with an increase of the 3-methyl to desmethylated homologues ratio at 3% salinity (Table 2). Such increased production of 3-methyl compounds is possibly an adaptive response to more extreme conditions since 3-methylhopanoids were shown to play an important role in maintaining the cell membrane integrity under unfavorable conditions in mesocosm experiments (Osborne et al. 2017).

**Fig. 8:**
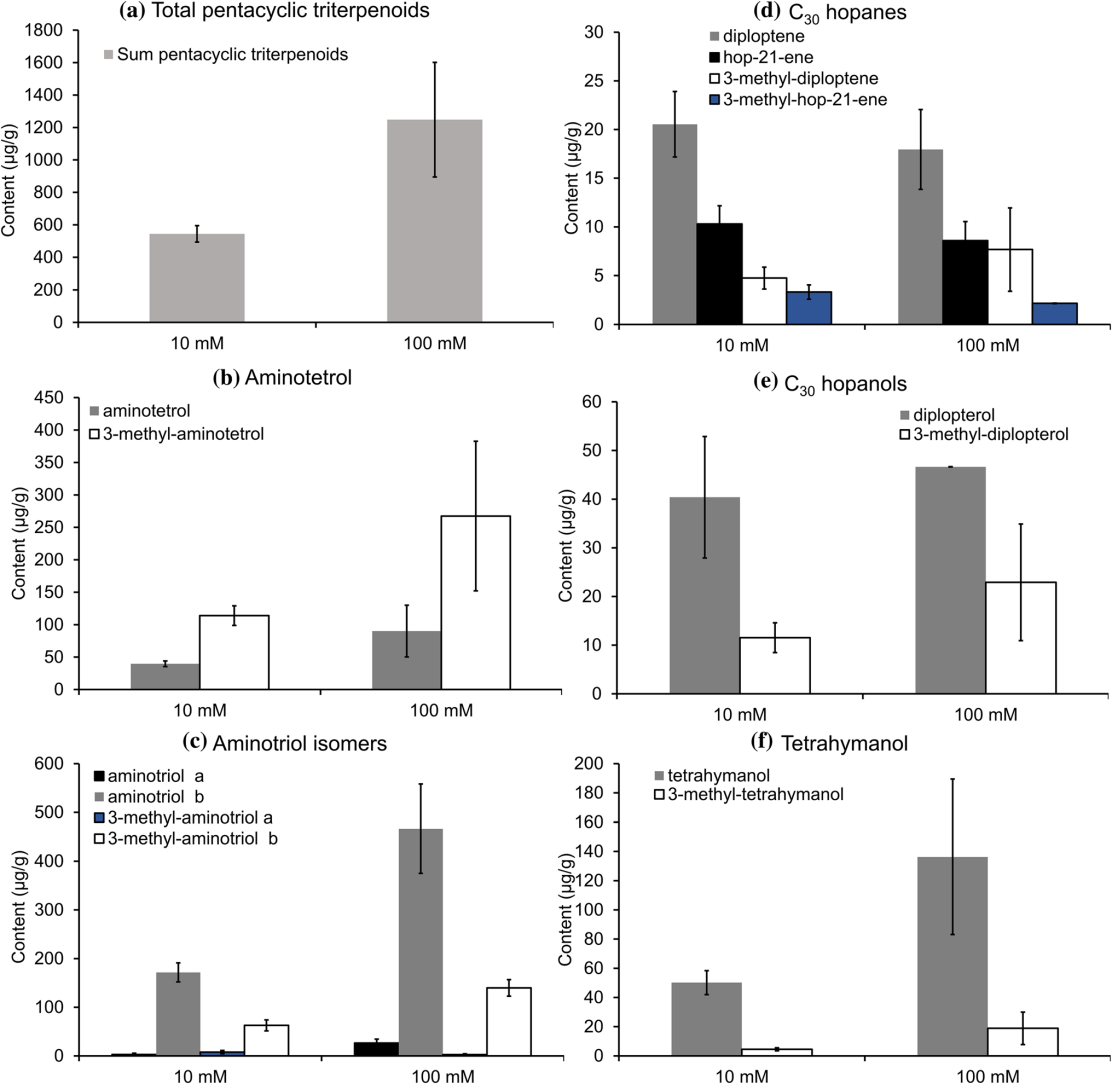
Distribution of pentacyclic triterpenoids in response to variations in nitrate concentration (10 mM and 100 mM): **a** total pentacyclic triterpenoids, **b** aminotetrol, **c** aminotriol isomers, **d** C_30_ hopanoids, **e** C_30_ hopanols, **f** tetrahymanol. BHPs were measured as products of periodic acid cleavage (aminotetrol yielded homohopanol, aminotriols yielded bishomohopanols). Error bars represent standard errors for *n* = 3 replicates per condition

In experiments with high nitrate level, the total contents of pentacyclic triterpenoids increased (Fig. 8a; Table 3). Particularly BHP contents increased (Fig. 8b, c), especially the aminotriol isomers **IIIa** and **b**, as well as 3-methyl-aminotriol b **IVb** (Fig. 8c). Contents of these compounds were more than doubled compared to standard culture conditions (Table 3, unpaired *t* test; *p* values from *t* tests provided in Supplementary File). For some compounds (namely hopanoids, hopanols, tetrahymanol, and aminotetrol), the standard deviation is unfortunately too high—even considering the natural variability typically associated to culture experiments—to make robust interpretations regarding the behavior of these lipids at different nitrate concentrations.

Considering the relative percentages of compounds, all BHPs showed an increase of 7%, regular aminotriol b **IIIb** increased by 8%, while relative contents of aminotetrol **V**, 3-methyl-aminotetrol **VI**, and 3-methyl-aminotriol b **IVb** remained similar at higher nitrate concentrations (not shown). This may suggest that higher BHP contents at higher nitrate concentration are especially related to increased production of aminotriol. To date, few studies have been undertaken to understand the role of nitrate in hopanoid production, specifically in hopanoid-producing bacteria capable of nitrogen fixation (*Frankia sp*., Nalin et al. 2000; *Nostoc punctiforme*, Doughty et al. 2009; *Desulfovibrio bastinii*, Blumenberg et al. 2012). For instance, experiments with strains of *Frankia sp.*, under nitrogen-enriched (NH_4_^+^) and nitrogen-depleted (nitrogen fixation) conditions, revealed that BHP contents in most of the strains were slightly higher under nitrogen-enriched conditions (Nalin et al. 2000). In the cyanobacterium *N. punctiforme*, nitrogen limitation enhanced BHP production as a survival mechanism during the first 2 weeks of incubation, returning afterwards to the starting levels encountered during standard conditions (Doughty et al. 2009). A similar study with the sulfate-reducing bacterium *D. bastinii*, grown with NH_4_^+^ or N_2_ as sole nitrogen source, found that nitrogen conditions do not much affect hopanoid composition, although the growth yield was higher with NH_4_ as nitrogen source (Blumenberg et al. 2012). The conflicting results of these studies argue against a direct link between hopanoid production and nitrogen uptake. Instead, hopanoid production is more likely related to physiological processes reflecting adaptation. Although we are only able to use some of the data of the nitrate experiments for a statistically sound comparison, the results of this study are in line with some of the earlier studies, documenting a strong positive response in BHP production to increased nitrate concentrations.

The ratio of 3-methyl to desmethyl homologues tended to increase slightly in high nitrate experiments too. This applies to C_30_ hopanoids, tetrahymanol, and aminotetrol as well, but this ratio decreased for aminotriol b (Table 2). The effect of the culture conditions on hopanoid methylation seems to have been a minor variable under the chosen conditions, although it has been shown that the degree of methylation influences membrane stability as well (Doughty et al. 2009; Welander et al. 2009). Future experiments on the factors governing lipid production by aerobic methanotrophs should expand the range of environmental conditions to better constrain the patterns in the response of methanotrophs to changing conditions.

## Conclusions

Besides previously reported BHPs, *M. alcaliphilum* was shown to synthesize four novel *N*-containing BHPs identified as aminodiol, 3-methyl-aminodiol, and early eluting isomers of aminotriol and 3-methyl-aminotriol. When grown at different salinities and nitrate concentrations, *M. alcaliphilum* strains revealed higher growth rates and shorter lag phases at lower salinity (1% NaCl) and higher nitrate concentration (100 mM). Our results also demonstrate an effect of salinity and nitrate concentration on the abundance and composition of pentacyclic triterpenoids. Hopanoid abundance was found to be higher in low salinity experiments (1% NaCl). Likewise, low salinity settings favored the production of BHPs, especially that of regular aminotriol b, while higher salinity (3% NaCl) favored the synthesis of tetrahymanol and 3-methyl-aminotetrol. Production of 3-methyl compounds was favored at higher salinity as well, reflected in an increase in the ratio of methylated to desmethylated compounds. In experiments with varying nitrate concentrations, higher concentrations correlated with more production of BHPs (aminotriol and 3-methyl-aminotriol).

### Supplementary Information

Below is the link to the electronic supplementary material.

Supplementary file1 (DOCX 67 KB)

## Appendix

See Fig. 9.
