# Supplementary material for: Variation of salinity and nitrogen concentration affects the pentacyclic triterpenoid inventory of the haloalkaliphilic aerobic methanotrophic bacterium Methylotuvimicrobium alcaliphilum
Source: Extremophiles. 2021 Apr 18;25(3):285–99. doi: 10.1007/s00792-021-01228-x (PMC8102298; doi:10.1007/s00792-021-01228-x)
Supplement: Supplementary file 1 — Supplementary file1 (DOCX 67 KB) [file 792_2021_1228_MOESM1_ESM.docx]

**Variations of nitrogen concentration and salinity affect the pentacyclic triterpenoid inventory of the haloalkaliphilic aerobic methanotrophic bacterium *Methylotuvimicrobium alcaliphilum***

Alexmar Cordova-Gonzalez^1^, Daniel Birgel^1^, Andreas Kappler^2^, and Jörn Peckmann^1^

**Supplementary information**

Supplementary Table 1. Contents of pentacyclic terpenoids in cultures of *M. alcaliphilum* provided with varying amounts of nitrate

|  | 10 mM KNO_3_ | | | | |  | 100 mM KNO_3_ | | | | |
| --- | --- | --- | --- | --- | --- | --- | --- | --- | --- | --- | --- |
|  | Content (µg/g dw) | | | | |  | Content (µg/g dw) | | | | |
|  | alc-1 | alc-2 | alc-3 | Average | SD |  | alc-4 | alc-5 | alc-6 | Average | SD |
| diploptene **XIII** | 19 | 27 | 16 | 21 | 6 |  | 10 | 19 | 24 | 18 | 7 |
| hop-21-ene **XIV** | 10 | 14 | 7 | 10 | 3 |  | 5 | 10 | 11 | 9 | 3 |
| 3-me-diploptene **XV** | 4 | 7 | 4 | 5 | 2 |  | 3 | 4 | 16 | 8 | 7 |
| 3-me-hop-21-ene **XVI** | 2 | 4 | 4 | 3 | 1 |  | 2 | 2 | **11** | 2 | 0 |
| diplopterol **XVII** | 19 | 63 | 39 | 40 | 22 |  | 47 | **16** | 47 | 47 | 0 |
| 3-me-diplopterol **XVIII** | 7 | 17 | 11 | 12 | 5 |  | 17 | 6 | 46 | 23 | 21 |
| tetrahymanol **XIX** | 60 | 56 | 34 | 50 | 14 |  | 53 | 121 | 235 | 136 | 92 |
| 3-me-tetrahymanol **XX** | 6 | 5 | 2 | 4 | 2 |  | 5 | 11 | 41 | 19 | 19 |
| aminodiol **I** | 0 | 0 | 0 | 0 | 0 |  | 1 | 3 |  | 2 | 1 |
| aminotriol a **IIIa** | 0 | 0 | 9 | 3 | 5 |  | 15 | 24 | 41 | 27 | 14 |
| aminotriol b **IIIb** | 138 | 172 | 206 | 172 | 34 |  | 321 | 443 | 636 | 467 | 159 |
| 3-me-aminotriol a **IVa** | 6 | 14 | 3 | 8 | 6 |  | 3 | 5 |  | 4 | 2 |
| 3-me-aminotriol b **IVb** | 44 | 61 | 83 | 63 | 20 |  | 106 | 154 | 160 | 140 | 30 |
| aminotetrol **V** | 37 | 48 | 34 | 40 | 8 |  | 43 | 58 | 170 | 90 | 69 |
| 3-me-aminotetrol **VI** | 106 | 143 | 93 | 114 | 26 |  | 125 | 182 | 496 | 267 | 200 |
| Sum pentacyclic triterpenoids | 457 | 632 | 544 | 544 | 88 |  | 754 | 1042 | 1922 | 1239 | 608 |

Bold values represent outliers according to Dixon test (α<0.05)

Supplementary Table 2. Contents of pentacyclic terpenoids in cultures of *M. alcaliphilum* grown at different salinities

|  | 1% NaCl | | | | |  | 3% NaCl | | | | |
| --- | --- | --- | --- | --- | --- | --- | --- | --- | --- | --- | --- |
|  | Content (µg/g dw) | | | | |  | Content (µg/g dw) | | | | |
|  | alc-7 | alc-8 | alc-9 | Average | SD |  | alc-1 | alc-2 | alc-3 | Average | SD |
| diploptene **XIII** | 20 | 41 | 23 | 28 | 12 |  | 19 | 27 | 16 | 21 | 6 |
| hop-21-ene **XIV** | 13 | 23 | 13 | 16 | 6 |  | 10 | 14 | 7 | 10 | 3 |
| 3-me-diploptene **XV** | 3 | 9 | 4 | 6 | 3 |  | 4 | 7 | 4 | 5 | 2 |
| 3-me-hop-21-ene **XVI** | 2 | **5** | 2 | 2 | 0 |  | 2 | 4 | 4 | 3 | 1 |
| diplopterol **XVII** | 118 | 51 | 49 | 72 | 39 |  | 19 | 63 | 39 | 40 | 22 |
| 3-me-diplopterol **XVIII** | 16 | 14 | 14 | 15 | 1 |  | 7 | 17 | 11 | 12 | 5 |
| tetrahymanol **XIX** | 53 | 58 | 55 | 55 | 2 |  | 60 | 56 | 34 | 50 | 14 |
| 3-me-tetrahymanol **XX** | 2 | 3 | 1 | 2 | 1 |  | 6 | 5 | 2 | 4 | 2 |
| aminodiol **I** | 14 | 7 | 16 | 12 | 4 |  | 0 | 0 | 0 | 0 | 0 |
| aminotriol a **IIIa** | 10 | 8 | 9 | 9 | 1 |  | 0 | 0 | 9 | 3 | 5 |
| aminotriol b **IIIb** | 441 | 652 | 168 | 420 | 243 |  | 138 | 172 | 206 | 172 | 34 |
| 3-me-aminotriol a **IVa** | 96 | 41 | 77 | 71 | 28 |  | 6 | 14 | 3 | 8 | 6 |
| 3-me-aminotriol b **IVb** | 106 | 192 | 52 | 117 | 70 |  | 44 | 61 | 83 | 63 | 20 |
| aminotetrol **V** | 110 | 132 | 38 | 93 | 49 |  | 37 | 48 | 34 | 40 | 8 |
| 3-me-aminotetrol **VI** | 119 | 182 | 78 | 127 | 52 |  | 106 | 143 | 93 | 114 | 26 |
| Sum pentacyclic triterpenoids | 1123 | 1414 | 599 | 1045 | 413 |  | 457 | 632 | 544 | 544 | 88 |

Bold values represent outliers according to Dixon test (α<0.05)

Supplementary Table 3. Calculated *p* values from *t*-tests

|  | Nitrogen availability |  | Salinity |
| --- | --- | --- | --- |
|  | 10 mM vs. 100 mM |  | 1% vs. 3% |
| diploptene **XIII** | 0.651 |  | 0.374 |
| hop-21-ene **XIV** | 0.562 |  | 0.196 |
| 3-me-diploptene **XV** | 0.543 |  | 0.738 |
| 3-me-hop-21-ene **XVI** | 0.497 |  | 0.414 |
| diplopterol **XVII** | 0.219 |  | 0.283 |
| 3-me-diplopterol **XVIII** | 0.409 |  | 0.409 |
| tetrahymanol **XIX** | 0.185 |  | 0.603 |
| 3-me-tetrahymanol **XX** | 0.267 |  | 0.071 |
| aminotriol a **IIIa** | **0.048** |  | 0.168 |
| aminotriol b **IIIb** | **0.035** |  | 0.251 |
| 3-me-aminotriol a **IVa** | 0.257 |  | **0.018** |
| 3-me-aminotriol b **IVb** | **0.020** |  | 0.269 |
| aminotetrol **V** | 0.336 |  | 0.201 |
| 3-me-aminotetrol **VI** | 0.318 |  | 0.729 |
| Sum pentacyclic triterpenoids | 0.236 |  | 0.224 |

Bold values represent those variables showing statistically significant differences (α<0.05)


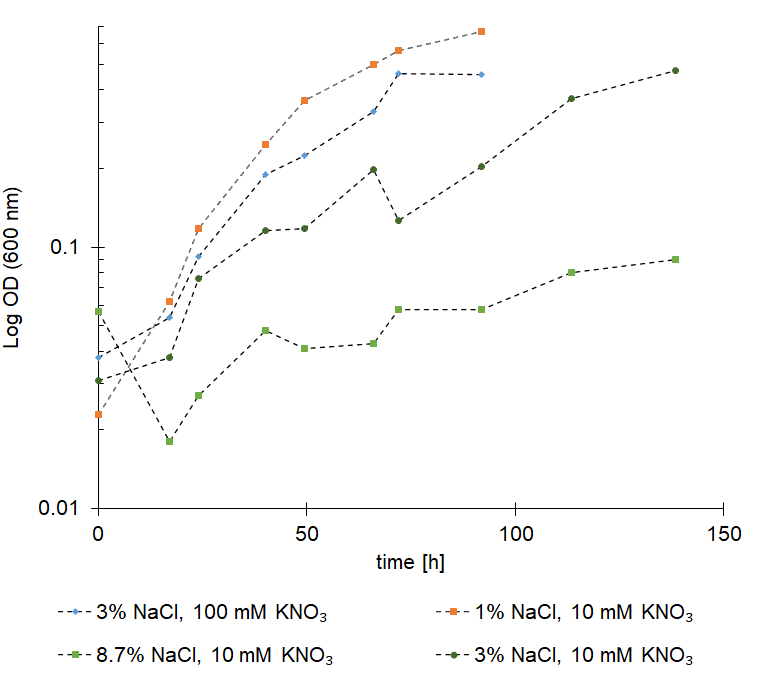


Supplementary Fig. 1. Growth curves (OD_600_) of *M. alcaliphilum* provided with varying amounts of nitrate and grown at different salinities.
